# High-Power Characteristics of Piezoelectric Transducers Based on [011] Poled Relaxor-PT Single Crystals

**DOI:** 10.3390/s25030936

**Published:** 2025-02-04

**Authors:** Soohyun Lim, Yub Je, Min-Jung Sim, Hwang-Pill Kim, Yohan Cho, Yoonsang Jeong, Hee-Seon Seo

**Affiliations:** 1 Agency for Defense Development, Changwon 51678, Republic of Korea; imsh@add.re.kr (S.L.); sideze@add.re.kr (Y.J.); mjsim@add.re.kr (M.-J.S.); pollux345@add.re.kr (H.-P.K.); yhcho@add.re.kr (Y.C.); 2 iBULe Photonics Co., Ltd., Incheon 21999, Republic of Korea; ysjeong@ibule.com

**Keywords:** piezoelectrics, ferroelectrics, relaxor-PbTiO3, Mn:PIN-PMN-PT, [011] poled single crystals, high-power characteristics, high-power transducers, flextensional transducer, underwater transducer

## Abstract

[011] poled relaxor-PT single crystals provide superior piezoelectric constants and electromechanical coupling factors in the 32 crystal directions, and also exhibit high electrical stability under compressive stresses and temperature changes. In particular, Mn-doped Pb(In_1/2_Nb_1/2_)O_3_-Pb(Mg_1/3_Nb_2/3_)O_3_-PbTiO_3_ (Mn:PIN-PMN-PT) single crystals show a superior coercive field (*E*_C_ ≥ 8.0 kV/cm) and mechanical quality factor (*Q*_m_ ≥ 1030), making them suitable for high-power transducers. The high-power characteristics of [011] poled single crystals have been verified from a material perspective; thus, further investigation is required from a transducer perspective. In this study, the high-power characteristics of piezoelectric transducers based on [011] poled PIN-PMN-PT and [011] poled Mn:PIN-PMN-PT single crystals were investigated. To analyze the driving limits of the single crystals, the polarization–electric field (P–E) curves, as a function of the driving electric field, were measured. The results showed that [011] poled Mn:PIN-PMN-PT single crystals demonstrate lower energy loss and THD (Total Harmonic Distortion), directly relating to the driving efficiency and linearity of the transducer. Additionally, [011] poled Mn:PIN-PMN-PT crystals provide excellent stability under the compressive stress and temperature changes. To analyze the high-power characteristics of [011] poled single-crystal transducers, two types of barrel-stave transducers, based on [011] poled PIN-PMN-PT and [011] poled Mn:PIN-PMN-PT, were designed and fabricated. The changes in the impedance and transmitting voltage response with respect to the driving electric fields were measured, and the energy loss and THD of the transducers with respect to the driving electric fields were examined to assess the driving limit of the [011] poled single-crystal transducer. The high-power characteristic tests confirmed the stability of [011] poled Mn:PIN-PMN-PT single crystals and verified their potential for high-power transducer applications.

## 1. Introduction

Relaxor-PT single crystals, exhibiting superior piezoelectric properties compared to conventional Pb(ZrTi)O_3_ (PZT) ceramics, are extensively studied for application in underwater sonar transducers and medical diagnostic transducers. Since relaxor-PT single crystals, e.g., Pb(Mg_1/3_Nb_2/3_)O_3_-PbTiO (PMN-PT), Pb(In_1/2_Nb_1/2_)O_3_-Pb(Mg_1/3_Nb_2/3_)O_3_-PbTiO_3_ (PIN-PMN-PT), and Pb(Mg_1/3_Nb_2/3_)O_3_-PbZrO_3_-PbTiO_3_ (PMN-PZT), provide high piezoelectric constants (*d*_33_ ≥ 1500 pC/N) and electromechanical coupling factors (*k*_33_ ≥ 0.90), single crystals can achieve a broad bandwidth and high sensitivity when they are used in piezoelectric transducers. Furthermore, their high elastic compliance enables the miniaturization and lightweighting of piezoelectric transducers [1,2,3,4].

Despite their superior piezoelectric properties, single crystals have been limited in their use to high-power transducers because of the constraints on their operating temperature and driving electric field. Considerable research has been conducted to address these constraints by increasing the rhombohedral–orthorhombic (R-O) phase transition temperature (*T*_RT_ ~ 80 °C) and coercive field (*E*_C_). First-generation crystals, represented by PMN-PT, exhibit high piezoelectric constants, but their rather low phase transition temperature (*T*_RT_ ~ 80 °C) and coercive field (*E*_C_ ~ 2.5 kV/cm) restrict the usable ranges of the temperature and driving field. Second-generation crystals, such as PIN-PMN-PT and PMN-PZT, have demonstrated improvements in the phase transition temperature (*T*_RT_ ~ 110 °C) and coercive field (*E*_C_ ~ 5.0 kV/cm). Recently developed third-generation crystals such as Mn:PIN-PMN-PT and Mn:PMN-PZT exhibit further increases in their phase transition temperature (*T*_RT_ ~ 120 °C), coercive field (*E*_C_ ~ 6.0 kV/cm), and mechanical quality factor (*Q*_m_ ~ 800), confirming their suitability for high-power transducer applications [5,6,7,8,9].

For piezoelectric transducers, single crystals with a rhombohedral phase, capable of spontaneous polarization in eight <111> directions, are mostly used. These rhombohedral phase crystals exhibit different piezoelectric properties depending on the polarization direction, and single crystals poled along the [001], [011], and [111] directions are preferred due to their high piezoelectric constant [5,10]. [111] poled single crystals have macroscopic 3 m symmetry, high piezoelectric constants, and electromechanical coupling factors in the 15 and 24 crystal directions. However, utilizing and fabricating shear-mode piezoelectric drivers of 15 and 24 modes is rather challenging. [001] poled single crystals, having a macroscopic 4 mm symmetry, are characterized by high piezoelectric constants and electromechanical coupling factors in the 33 crystal direction. Since the identical direction of the driving field (3) and the deformation (3) facilitate the design and manufacture of the piezoelectric driver, most single crystal transducers use the piezoelectric driver based on [001] poled single crystals [11,12]. However, under high-power driving conditions, such as an excessive driving field, mechanical stress or temperature, [001] poled single crystals show a decrease in the coercive field and R-O phase transition [13,14]. Uni-polar driving with a DC bias voltage can address this issue [15], but additional driving electronics are required.

[011] poled single crystals, having a macroscopic mm2 symmetry, provide high piezoelectric constants and electromechanical coupling factors in the 32 crystal directions. [011] poled single crystals driven in the 32 vibration mode demonstrate high electrical stability because the driving electric field direction (3) and the deformation direction (2) are orthogonal, ensuring the independence of the electric field and deformation. Furthermore, recent studies have indicated that third-generation [011] poled Mn:PIN-PMN-PT single crystals offer a superior coercive field (*E*_C_ ≥ 8.0 kV/cm) and mechanical quality factor (*Q*_m_ ≥ 1030) compared to single crystals with other domain engineering [16]. Numerous studies have investigated the driving field limits and phase transition temperature characteristics of [011] poled single crystals with respect to various material compositions [17]. [011] poled single crystals have also demonstrated high stability and driving linearity under compressive stress induced by pre-stress and heat generated by high-power driving [18,19]. In terms of single-crystal transducers, K. A. Snook et al. reported analytic and numerical studies on a performance comparison between a 33-mode tonpilz transducer based on [001] poled single crystals and a 32-mode tonpilz transducer based on [011] poled single crystals [20,21]. Additionally, K. Zhang et al. fabricated a 32-mode tonpilz transducer based on [011] poled single crystals and evaluated its primary acoustic characteristics [22]. Research on piezoelectric transducers based on [011] poled single crystals has not been performed extensively due to the challenges in designing and fabricating 32-vibration-mode piezoelectric drivers. Therefore, further investigation is required to explore the use of [011] poled single crystals with high electrical stability in high-power transducers.

In this study, the high-power characteristics of [011] poled single crystals with two compositions, PIN-PMN-PT and Mn:PIN-PMN-PT, were investigated. From the material perspective, [011] poled PIN-PMN-PT and [011] poled Mn:PIN-PMN-PT specimens were fabricated, and polarization–electric field (P–E) curves were measured as a function of the driving electric field to investigate their nonlinear characteristics. To examine the influence of the high-power characteristics of the materials on the transducer performance, two types of transducer, each utilizing a piezoelectric driver based on [011] poled PIN-PMN-PT and [011] poled Mn:PIN-PMN-PT, were fabricated. The acoustic characteristics were measured as a function of the driving electric field to further analyze and compare the nonlinearity characteristics.

In Section 2, to analyze the driving limits of [011] poled PIN-PMN-PT and [011] poled Mn:PIN-PMN-PT single crystals, the P–E curves, as a function of the driving electric field, were measured, and the energy loss and THD (Total Harmonic Distortion) with an increasing driving electric field were examined. Additionally, the energy loss under compressive stress and temperature conditions was examined to investigate the effects of external stress and thermal variations in the single-crystal transducers. In Section 3 and Section 4, to analyze the high-power driving characteristics of [011] poled single-crystal transducers, two types of barrel-stave transducers, based on [011] poled PIN-PMN-PT and [011] poled Mn:PIN-PMN-PT, were designed and fabricated. The transducer converts the longitudinal vibration of the piezoelectric driver, comprising single-crystal stacks driven in 32 vibration modes, into horizontal vibration through concave radiating plates positioned along the side of the piezoelectric driver to radiate sound waves [23,24]. The in-water impedance and transmitting voltage response of the fabricated single-crystal transducer were measured in Lake test facilities. The changes in the impedance and transmitting voltage response with respect to driving electric fields were measured, and the energy loss and THD of the transducers, as a function of the driving electric field, were examined to assess the driving limit of the [011] poled single-crystal transducer.

## 2. Field-Dependent Test of [011] Poled Single Crystals

[011] poled single crystals, exhibiting high piezoelectric constants in the 32 crystal direction, are suitable for 32-mode operation. The orthogonal relationship between the driving electric field direction (3) and the deformation direction (2) ensures high stability under high-power driving [16,18]. To confirm the high-power characteristics of [011] poled piezoelectric single crystals in terms of the material itself, two types of specimens, [011] poled PIN-PMN-PT and [011] poled Mn:PIN-PMN-PT, were fabricated. The P–E curves were measured by changing the driving electric field applied to the piezoelectric single crystals, and the energy loss and THD changes were calculated. Additionally, considering the driving conditions of high-power single-crystal transducers, the P–E curves and energy loss changes under varying compressive stress and temperature conditions were investigated.

### 2.1. Test Set-Ups

**Figure 1:**
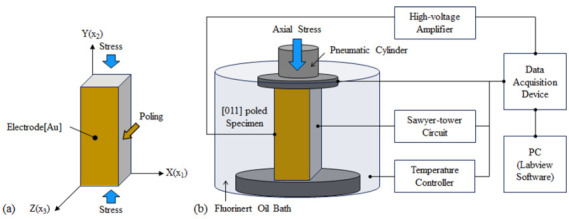
Set-ups for field-dependent test of [011] poled PIN-PMN-PT and [011] poled Mn:PIN-PMN-PT single crystals: (**a**) Schematic drawing of the test specimen and (**b**) stress-dependent electro-mechanical characterization system.

Figure 1a shows a schematic drawing of the single-crystal specimen used to verify the high-power characteristics. Two types of specimens, [011] poled PIN-PMN-PT and [011] poled Mn:PIN-PMN-PT, grown by the Bridgman method in iBULe Photonics Inc., Incheon, Republic of Korea, were fabricated. The doped level of Mn was in the range of 1–5 mol. %. The Cr/Au electrodes (10 nm/100 nm) were deposited to polarize the specimens in the [011] direction. The dimensions of the specimens were designed to be 4 × 12 × 4 (X × Y × Z) mm^3^ so that 32 vibration modes could be dominantly excited. Figure 1b shows the configuration of the SDECS (Stress Dependent Electromechanical Characterization System, iBULe Photonics Inc., Incheon, Republic of Korea) used to measure the polarization as a function of the driving electric field while varying the compressive stress and temperature conditions applied to the specimens. The specimens were installed in an oil bath within the test chamber for electrical insulation. The driving electric field, in the form of a triangular wave generated by a data acquisition device (NI USB-6211, NI, Austin, USA), was amplified by a high-voltage amplifier (Model 20, Advanced Energy industries Inc., Lockport, USA) and applied to the specimen to drive it. The driving current was measured using a Sawyer–Tower circuit connected to the specimen, and the polarization of the specimen, as a function of the electric field, was calculated and stored via the data acquisition device. The pneumatic cylinder was used to apply the necessary compressive stress (2 to 40 MPa) in the specimen’s longitudinal direction (2), and a temperature controller attached to the test chamber was used to apply the necessary temperature (5 to 60 °C).

### 2.2. Test Results

**Figure 2:**
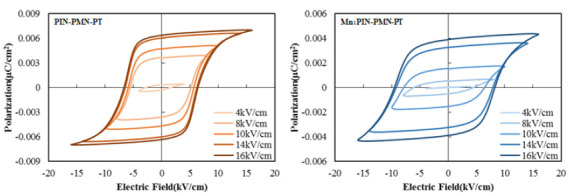
Polarization–electric field with respect to the different driving electric field of [011] poled PIN-PMN-PT and [011] poled Mn:PIN-PMN-PT.

Figure 2 presents the polarization–electric field with respect to the different driving electric fields of [011] poled PIN-PMN-PT and [011] poled Mn:PIN-PMN-PT. A compressive stress of 10 MPa and a temperature of 5 °C were applied to the specimens to match the pre-stress and test temperature of the fabricated transducer. The polarization was offset such that the center value was zero. The coercive field (*E*_C_), defined as the electric field at which polarization is reversed by the driving electric field, determines the limit of the driving electric field. When the driving electric field is sufficiently smaller than the coercive field, the electric field and polarization exhibit a linear relationship, and hysteresis does not occur. However, when the driving electric field approaches or exceeds the coercive field, significant hysteresis characteristics are observed.

The coercive fields of [011] poled PIN-PMN-PT and [011] poled Mn:PIN-PMN-PT single crystals were measured as 6.5 kV/cm and 9.0 kV/cm, respectively. This indicates that [011] poled Mn:PIN-PMN-PT single crystals can be driven with higher electric fields than [011] poled PIN-PMN-PT single crystals. In particular, [011] poled Mn:PIN-PMN-PT single crystals exhibit much higher coercive fields compared to the [001] poled single crystals, which have coercive fields of 5.0 kV/cm and 6.0 kV/cm for Mn:PIN-PMN-PT and PIN-PMN-PT single crystals, respectively [2,5]. Therefore, [011] poled single crystals (especially Mn:PIN-PMN-PT single crystals) can provide high linearity under high electric fields compared to the [001] poled single crystals.

**Figure 3:**
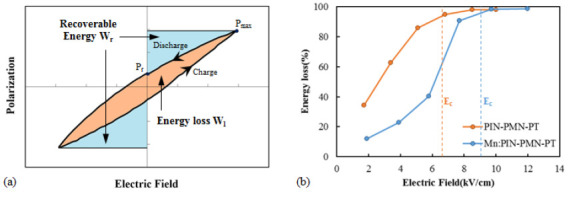
(**a**) Polarization–electric field hysteresis loop of single crystals and (**b**) energy loss as a function of the driving electric field.

Figure 3a shows a typical P–E curve hysteresis loop of a piezoelectric single crystal, where the inner area of the hysteresis loop represents the energy loss (*W*_l_) and the outer area represents the recoverable energy (*W*_r_) [25,26,27]. The energy loss of single crystals in the hysteresis loop is defined by Equation (1).

(1) $$Energy\;loss(\%)=\frac{W_{l}}{W_{r}+W_{l}}\times 100\%$$

Figure 3b presents the energy loss as a function of the driving electric field for [011] poled PIN-PMN-PT and [011] poled Mn:PIN-PMN-PT, calculated using Equation (1). As the driving electric field increases, energy loss, comprising the dielectric loss from polarization rotation and the mechanical loss dissipated into heat significantly, increases [9,28,29]. Specifically, the energy loss exceeds 90% when the driving electric field is above the coercive field. The [011] poled Mn:PIN-PMN-PT single crystal with a higher coercive field results in half the energy loss of [011] poled PIN-PMN-PT at the same driving electric field. As the energy loss of a piezoelectric single crystal directly relates the electro-acoustic efficiency of transducers, the efficiency under high-power driving needs to be investigated for the verification of the high-power characteristics of single-crystal transducers.

**Figure 4:**
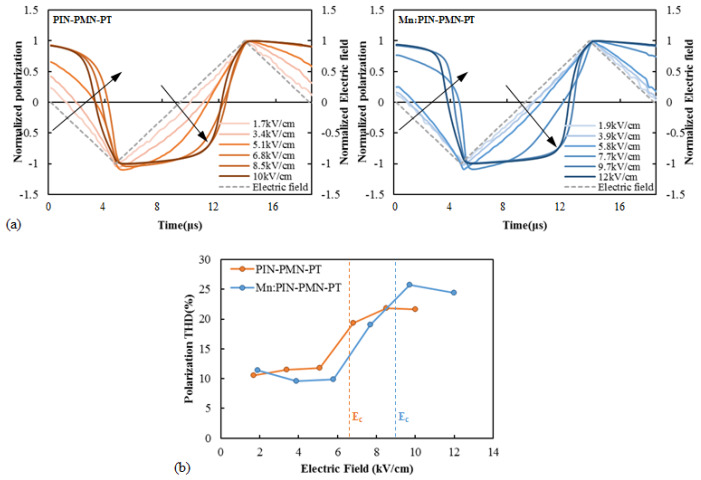
(**a**) Polarization waveform with respect to the different driving electric field and (**b**) total harmonic distortion of polarization as a function of the driving electric field of [011] poled PIN-PMN-PT and [011] poled Mn:PIN-PMN-PT.

Figure 4 presents (a) the normalized waveform of the polarization with respect to the different driving electric field of [011] poled PIN-PMN-PT and [011] poled Mn:PIN-PMN-PT, and (b) their THD calculated by FFT analysis. When the driving electric field is significantly smaller than the coercive field, the polarization waveform and the driving electric field’s time waveform are linear. However, as the driving electric field increases, nonlinear distortion appears in the polarization waveform over time, as indicated by the black arrows in the figures. The THD of the polarization waveform under a small driving electric field is approximately 12%, which is the general THD value of the triangular wave [30,31]. However, the THD increases under a high driving electric field, exceeding 20% when the driving electric field is above the coercive field. [011] poled Mn:PIN-PMN-PT single crystals with a higher coercive field exhibit a lower THD than [011] poled PIN-PMN-PT under the same driving electric field. As the THD of a piezoelectric single crystal directly relates to the transducer’s distortion of the driving current and the generated sound wave, the THD of the transducers under high-power driving needs to be investigated for the verification of the high-power characteristics of single-crystal transducers.

For piezoelectric transducers operating with high power, pre-stress is typically required to prevent the fracture of single crystals by tensile dynamic stress. Additionally, temperature increases caused by heat generation during high-power driving are inevitable. Therefore, verifying the characteristic changes in the single crystal under compressive stress and temperature conditions is crucial for their application in high-power transducers. For the [011] poled PIN-PMN-PT and [011] poled Mn:PIN-PMN-PT single crystals under varying compressive stress and temperature conditions, P–E curves were measured for different compressive stresses (2, 10, 20, 30, 40 MPa) and temperatures (5, 20, 40, 60 °C), and the energy loss was calculated using Equation (1).

**Figure 5:**
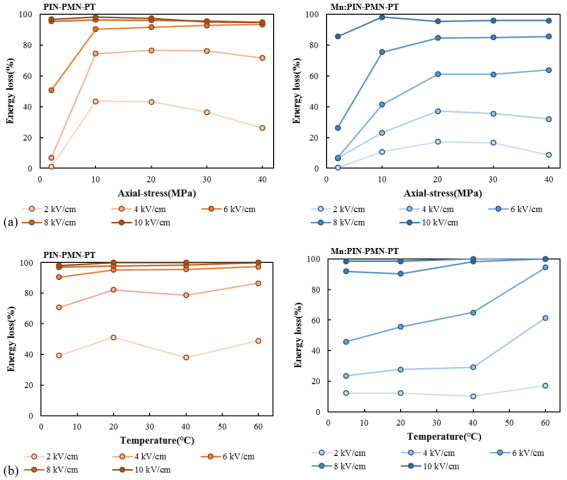
Energy loss as a function of the driving electric field with axial-stress changes (**a**) and temperature changes (**b**) for [011] poled PIN-PMN-PT and [011] poled Mn:PIN-PMN-PT.

Figure 5a presents the energy loss of [011] poled PIN-PMN-PT and [011] poled Mn:PIN-PMN-PT single crystals with various compressive stress conditions (2, 10, 20, 30, 40 MPa). As the applied compressive stress increases, the energy loss of the single crystal increases, and this trend converges when the compressive stress exceeds 20 MPa. As the compressive stress distorts the crystal structure and polarization directions and finally the resulting R–O phase transition, the single crystal under compressive stress thus exhibits high energy loss [18]. Figure 5b presents the energy loss of [011] poled PIN-PMN-PT and [011] poled Mn:PIN-PMN-PT single crystals with temperature changes (5, 20, 40, 60 °C). As the driving temperature increases under the same driving electric field, the energy loss of the single crystal increases by over 90% when driven above the coercive field. The temperature increase also accelerates the R–O phase transition and results in an increase in energy loss for the [011] poled single crystal [18].

The previous measurements show that [011] poled single crystals, as is well known, exhibit nonlinearity and hysteresis in their response due to polarization rotation and reversal above the coercive field. Notably, even when driven below the coercive field, a significant amount of energy loss and distortion still occurs. In particular, as [011] poled Mn:PIN-PMN-PT single crystals exhibit a much higher coercive field compared to not only [011] poled PIN-PMN-PT and but also [001] poled single crystals, [011] poled Mn:PIN-PMN-PT single crystals can provide great advantages in linearity and efficiency for high-power transducers.

## 3. Design and Fabrication of the Transducer

Two types of barrel-stave transducers, based on [011] poled PIN-PMN-PT and [011] poled Mn:PIN-PMN-PT, were designed and fabricated to verify the high-power characteristics of [011] poled single crystal transducers. The barrel-stave transducer converts the longitudinal vibration generated by the piezoelectric driver into the flexural vibration of a radiating plate in order to radiate low-frequency sound. Class I barrel-stave transducers address the concave radiating plates, and apply compressive stress to the piezoelectric driver under hydrostatic pressure.

Flextensional transducers, such as barrel-stave transducers, have commonly utilized piezoelectric drivers with a [001] poled orientation due to their ease of design and fabrication [11,12]. [011] poled piezoelectric single-crystal drivers, driven in 32 vibration modes, are expected to be suitable for high-power transducer applications because of their high electrical stability, which results from the orthogonal relationship between the field direction (3) and the deformation direction (2) [20,21,22]. In this study, a piezoelectric single-crystal driver with a configuration suitable for 32 vibration modes and applicable to barrel-stave transducers was proposed, designed, and fabricated, and its high-power driving characteristics were evaluated.

**Figure 6:**
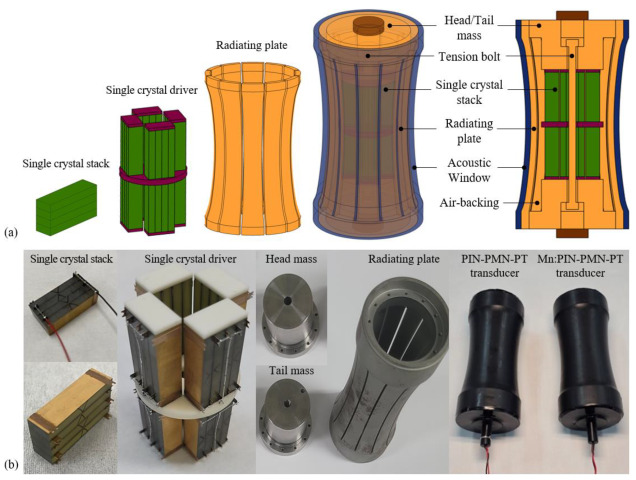
(**a**) Schematic drawing of the barrel-stave single crystal-transducer and (**b**) the fabricated barrel-stave transducers.

Figure 6a presents a schematic diagram of the barrel-stave single-crystal transducers. [011] poled PIN-PMN-PT and [011] poled Mn:PIN-PMN-PT single crystals grown by the Bridgman method in iBULe Photonics Inc., Incheon, Republic of Korea, were used for the piezoelectric elements. Single-crystal facets were designed with dimensions of 8 × 28 × 3.5 mm^3^ (X × Y × Z) for 32-vibration-mode operation with the desired resonance frequency. A single crystal stack comprises four single-crystal facets connected in parallel, and four piezoelectric stacks arranged into two layers to amplify longitudinal vibration. Insulating plates were applied to the upper and lower surfaces of the single crystal stacks to electrically isolate the high-voltage signal from the transducer structure. Head and Tail masses were attached to both ends of the piezoelectric driver, with steel radiating plates arranged and attached to the sides of these masses. To minimize the effects of hoop stress during the flexural mode vibration of the radiating plates, slits were added to divide the radiating plate into 12 sections. To prevent damage to the piezoelectric single crystals due to tensile stress during high-power driving, a compressive pre-stress of 12 MPa was applied using tension bolts. An EPDM acoustic window was applied to the transducer’s exterior for waterproofing. The piezoelectric single-crystal transducer has dimensions of D62 × H138 mm, with a resonance frequency designed at 1.55 kHz. Figure 6b shows the fabricated barrel-stave transducers [011] poled Mn:PIN-PMN-PT single crystals.

## 4. Experimental Results and Discussions

The acoustic characteristics of the fabricated single-crystal transducer were tested. The changes in admittance and TVR (transmitting voltage response) were measured with respect to the driving voltage amplitude of the transducer. The energy loss and THD of the transducers were calculated and evaluated from the measured data.

### 4.1. Experimental Set-Ups

**Figure 7:**
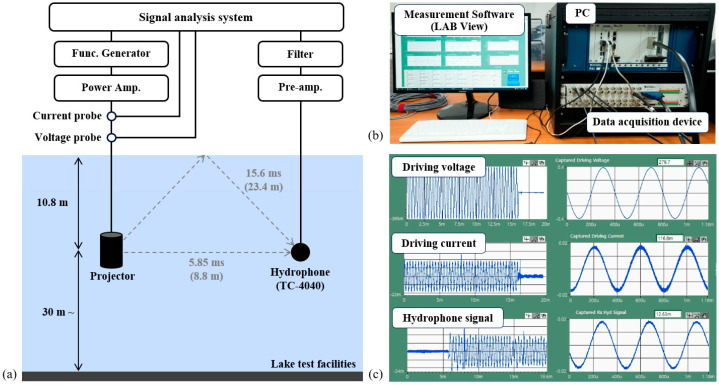
(**a**) Schematic diagram of the measurement setup, (**b**) the signal analysis system, and (**c**) the measurement software.

Underwater acoustic characteristic tests of the transducers were performed in Lake test facilities. Figure 7a shows a schematic diagram of the measurement setup. The fabricated relaxor-PT crystal transducer and a standard hydro-phone (TC4040, Teledyne Reson, Slangerup, Denmark) were installed at a water depth of 10.8 m with a distance of 8.8 m to minimize interference from surface-reflected waves. The water temperature at the installation depth was measured to be approximately 5 °C. The driving signal generated by a function generator (33500B, Agilent Technologies Inc., Santa Clara, CA, USA) was amplified using a high-voltage amplifier (L20, Instrument Inc., San Diego, CA, USA) and applied to the transducer to emit sound waves. The acoustic signal measured by a standard hydrophone was amplified, filtered, and acquired by a signal analysis system. As shown in Figure 7b, the signal analysis system comprises a data acquisition device, a PC, and measurement LabVIEW 2020. The waveforms of the driving voltage signal, current signal, and hydrophone receiving signal are shown in Figure 7c.

### 4.2. Experimental Results

**Figure 8:**
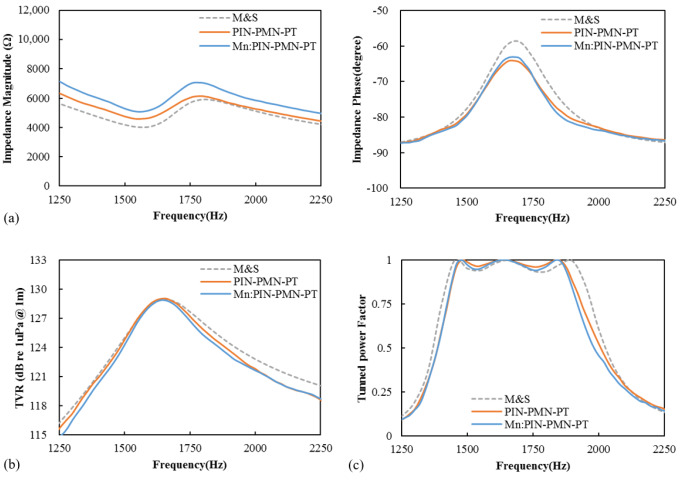
Measured (solid line) and simulated (dotted line) acoustic characteristics of [011] poled PIN-PMN-PT and [011] poled Mn:PIN-PMN-PT single-crystal transducers: (**a**) impedance magnitude and phase, (**b**) transmitting voltage response, and (**c**) series-tuned power factor.

Figure 8 presents the measured results (solid lines) and FEM analysis results (dotted lines) of the impedance, TVR, and power factor of the fabricated [011] poled PIN-PMN-PT and [011] poled Mn:PIN-PMN-PT single-crystal transducers. The measured results were obtained at a driving electric field of 0.4 kV/cm (corresponding to a driving voltage of 100 V_rms_). The simulated results were obtained via finite element analysis using the commercial software COMSOL Multiphysics 6.1. For the simulation, the same material properties of [011] poled PIN-PMN-PT and [011] poled Mn:PIN-PMN-PT were applied because the properties of the PIN-PMN-PT and Mn:PIN-PMN-PT facets from iBULe Photonics Inc., Republic of Korea, that were used for the fabrication of the transducers are similar [32,33]. Two fabricated transducers also exhibit similar acoustic characteristics. The measured impedance, TVR, and power factor results showed good agreement with the simulated results, validating the design performance of the fabricated transducers. The resonance frequencies of the fabricated [011] poled PIN-PMN-PT and [011] poled Mn:PIN-PMN-PT single-crystal transducers were identical at 1.56 kHz, with coupling coefficients of 0.50 and 0.49, respectively. The maximum TVR at the resonance frequency for both [011] poled PIN-PMN-PT and [011] poled Mn:PIN-PMN-PT single-crystal transducers was 129 dB, and the −3 dB bandwidths were 258 Hz and 228 Hz, respectively. The power factor of the transducers was tuned to zero, the reactance current at resonance was detected by connecting series inductors, and the half-power bandwidth of the transducers was 626 Hz and 616 Hz, respectively.

**Figure 9:**
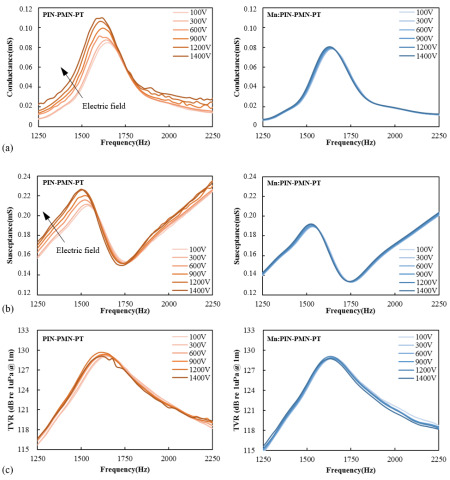
Measured conductance curve (**a**), susceptance curve (**b**), and transmitting voltage response (**c**) with respect to the different driving voltage of [011] poled PIN-PMN-PT and [011] poled Mn:PIN-PMN-PT single-crystal transducers.

Figure 9 presents the high-power characteristics of the fabricated [011] poled PIN-PMN-PT and [011] poled Mn:PIN-PMN-PT single-crystal transducers measured in the driving field range of 0.40–5.7 kV/cm (corresponding to a driving voltage of 100 V_rms_–1400 V_rms_). The maximum driving voltage of 1400 V_rms_ was limited by the maximum available voltage of the amplifier. The conductance and susceptance representing the resistance and reactance components of the transducer driving current, respectively, were related to the power consumption and recoverable power of the transducer. For the [011] poled PIN-PMN-PT single-crystal transducer, it was shown that as the driving electric field increased, the conductance and susceptance increased, thereby increasing the driving power per unit voltage, while the TVR remained constant. In contrast, for the [011] poled Mn:PIN-PMN-PT single-crystal transducer, it was observed that the conductance, susceptance, and TVR remained constant even as the driving electric field increased within the tested range.

To investigate the change in energy loss of the transducers with respect to the driving electric field under high-power driving, the radiated acoustic power (*P_acous_*), input electric power (*P_ele_*), and electro-acoustic energy loss were calculated from the measured conductance, susceptance, and TVR shown in Figure 9, using Equations (2)–(4) [34,35].

(2) $$P_{acous}={log}^{-1}\left(\frac{TVR-20\log\left(V\right)-DI-170.8\;dB}{10}\right)$$

(3) $$P_{elec}=GV^{2}$$

(4) $$Energy\;loss(\%)=\frac{P_{elec}-P_{acous}}{P_{elec}}\times 100\%$$

**Figure 10:**
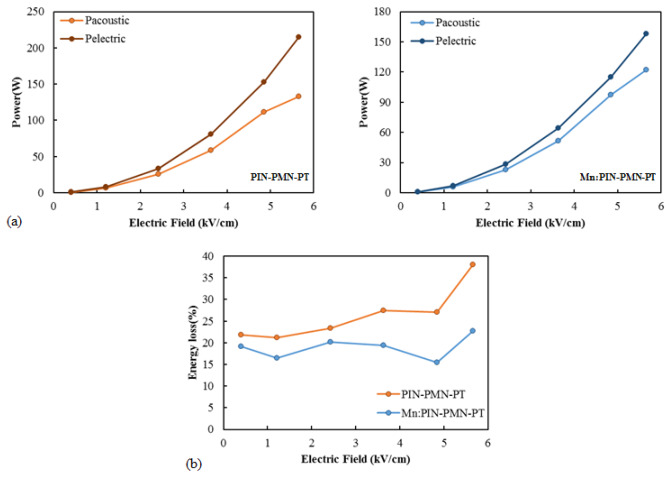
Acoustic and electric power (**a**) and energy loss (**b**) as a function of the driving electric field of [011] poled PIN-PMN-PT and [011] poled Mn:PIN-PMN-PT single-crystal transducers.

Here, *G* is conductance, *V* is the driving voltage, and *DI* is the directivity index. Since the fabricated transducer’s size is significantly smaller than the wavelength of the driving frequency, *DI* was approximated as 0. Figure 10a illustrates *P_acous_* and *P_ele_* as a function of the driving electric field of [011] poled PIN-PMN-PT and [011] poled Mn:PIN-PMN-PT single-crystal transducers. It was shown that both the input electric power and the radiated acoustic power increased as the driving electric field increased.

Figure 10b presents the energy loss of [011] poled PIN-PMN-PT and [011] poled Mn:PIN-PMN-PT single-crystal transducers as a function of the driving electric field. For [011] poled PIN-PMN-PT single-crystal transducer, the energy loss was measured at approximately 20% under low driving electric fields. However, as the driving electric field increased, the difference between the input electric power and the radiated acoustic power increased, leading to an energy loss exceeding 35%. In contrast, for the [011] poled Mn:PIN-PMN-PT single-crystal transducer, the energy loss remained constant around 20%, even as the driving electric field increased. The energy loss of the single-crystal facets in Figure 3 shows that [011] poled PIN-PMN-PT single crystals exhibit loss increase at electric fields above 2 kV/cm, while [011] poled Mn:PIN-PMN-PT single crystals exhibit loss increase at electric fields above 5 kV/cm. A similar trend was shown in the energy loss of the single-crystal transducer based on [011] poled crystals in Figure 10.

**Figure 11:**
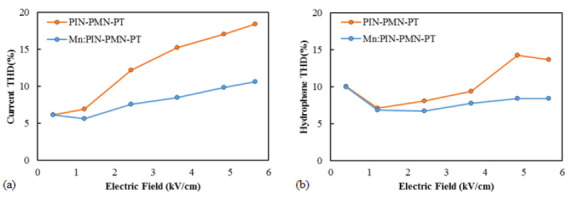
(**a**) Measured current total harmonic distortion–electric field response and (**b**) measured hydrophone total harmonic distortion–electric field response of [011] poled PIN-PMN-PT and [011] poled Mn:PIN-PMN-PT single-crystal transducers.

To evaluate the linearity of the fabricated transducers under high-power driving, the power spectrum of the driving current and the hydrophone receiving signal were measured, and the THD as a function of the driving electric field was calculated. Figure 11a presents the driving current THD of the [011] poled PIN-PMN-PT and [011] poled Mn:PIN-PMN-PT single-crystal transducers as a function of the driving electric field. As the driving field increased, the current THD of the [011] poled PIN-PMN-PT single-crystal transducer increased nearly threefold, from 6% at 0.4 kV/cm to 18% at 5.7 kV/cm. In contrast, the current THD of the [011] poled Mn:PIN-PMN-PT single-crystal transducer increased less than twofold, from 6% at 0.4 kV/cm to 10% at 5.7 kV/cm. Figure 11b presents the hydrophone THD of the [011] poled PIN-PMN-PT and [011] poled Mn:PIN-PMN-PT single-crystal transducers as a function of the driving electric field. As the driving electric field increased, the hydrophone THD of the [011] poled PIN-PMN-PT single-crystal transducer increased, whereas the hydrophone THD of the [011] poled Mn:PIN-PMN-PT single-crystal transducer remained constant. The polarization THD results for the single crystals in Figure 4 showed that [011] poled Mn:PIN-PMN-PT single crystals exhibited a lower THD than [011] poled PIN-PMN-PT single crystals at the same electric field, which aligns with the driving current and hydrophone THD results for the transducer in Figure 11.

The acoustic characteristics of the [011] poled PIN-PMN-PT and [011] poled Mn:PIN-PMN-PT single-crystal transducers were measured with respect to the driving electric field magnitude. The energy loss and distortion of the transducer based on [011] poled PIN-PMN-PT, which exhibited higher energy loss and distortion from the material perspective, were observed to be greater than those of the [011] poled Mn:PIN-PMN-PT single-crystal transducer. Notably, for the transducer based on [011] poled Mn:PIN-PMN-PT, the rate of increase in energy loss and distortion with respect to the driving electric field was low, and it was observed that high-power characteristics remained stable within the driving electric field range of 0.40–5.7 kV/cm (corresponding to a driving voltage of 100 V_rms_–1400 V_rms_).

## 5. Conclusions

In this study, the high-power driving characteristics of the piezoelectric transducers based on the [011] poled relaxor-PT single crystal were investigated. The P–E curves of the [011] poled PIN-PMN-PT and [011] poled Mn:PIN-PMN-PT single crystals were analyzed as a function of the driving electric field magnitude. The results showed that [011] poled Mn:PIN-PMN-PT single crystals exhibit a significantly higher coercive field compared to not only [011] poled PIN-PMN-PT but also [001] poled single crystals. Therefore, [011] poled Mn:PIN-PMN-PT single crystals demonstrate lower energy loss and THD, directly relating to the driving efficiency and linearity of the transducer. [011] poled Mn:PIN-PMN-PT crystals provide excellent stability under the compressive stress and temperature changes. In terms of transducer, the transducer based on [011] poled single crystals also demonstrated electrical stability under high-power driving conditions. In particular, the transducer based on [011] poled Mn:PIN-PMN-PT single crystals provides sufficiently low distortion and energy loss under high-power driving. The high-power characteristic tests confirmed the stability of the [011] poled Mn:PIN-PMN-PT single crystals and verified its potential for high-power transducer applications.

## Data Availability

Data are contained within the article.

## References

[1] Zhang S., Li F., Jiang X., Kim J., Luo J., Geng X. (2015). Advantages and challenges of relaxor-PbTiO3 ferroelectric crystals for electroacoustic transducers—A review. Prog. Mater. Sci..

[2] Zhang S., Li F., Yu F., Jiang X., Lee H.-Y., Luo J., Shrout T.R., Zhang S., Li F., Yu F. (2018). Recent developments in piezoelectric crystals. J. Korean Ceram. Soc..

[3] Ewart L.M., Mclaughlin E.A., Robinson H.C., Stace J.J., Amin A. (2007). Mechanical and electromechanical properties of PMNT single crystals for naval sonar transducers. IEEE Trans. Ultrason. Ferroelectr. Freq. Control.

[4] Sim M.-S., Je Y., Cho Y.-H., Seo H.-S., Kim M.-J. (2024). Derivation of equivalent material coefficients of 2-2 piezoelec-tric single crystal composite. Micromachines.

[5] Zhang S., Li F. (2012). High performance ferroelectric relaxor-PbTiO_3_ single crystals: Status and perspective. J. Appl. Phys..

[6] Zhang S., Li F., Shrout T.R., Luo J. Relaxor-PT single crystals for various applications. Proceedings of the ISAF-ECAPD-PFM 2012.

[7] Zhang S., Lee S.M., Kim D.H., Lee H.Y., Shrout T.R. (2008). Characterization of Mn-modified Pb (Mg1/3Nb2/3) O3–PbZrO3–PbTiO3 single crystals for high power broad bandwidth transducers. Appl. Phys. Lett..

[8] Sun E., Zhang S., Luo J., Shrout T.R., Cao W. (2010). Elastic, dielectric, and piezoelectric constants of Pb (In1/2Nb1/2) O3–Pb (Mg1/3Nb2/3) O3–PbTiO3 single crystal poled along [011] c. Appl. Phys. Lett..

[9] Zhang S., Xia R., Lebrun L., Anderson D., Shrout T.R. (2005). Piezoelectric materials for high power, high temperature applications. Mater. Lett..

[10] Zhang S., Sherlock N.P., Meyer R.J., Shrout T.R. (2009). Crystallographic dependence of loss in domain engineered relaxor-PT single crystals. Appl. Phys. Lett..

[11] Sherlock N.P., Meyer R.J. (2012). Modified single crystals for high-power underwater projectors. IEEE Trans. Ultrason. Ferroelectr. Freq. Control.

[12] Sherlock N.P. (2010). Relaxor-PT Single Crystals for Broad Bandwidth, High Power Sonar Projectors.

[13] Gallagher J.A., Tian J., Lynch C.S. (2014). Composition dependence of electro-mechanical properties and field induced phase transformations in [001] C PIN–PMN–PT single crystals. Smart Mater. Struct..

[14] Schader F.H., Rossetti Jr G.A., Luo J., Webber K.G. (2017). Piezoelectric and ferroelectric properties of< 001> C Pb (In1/2Nb1/2) O3-Pb (Mg1/3Nb2/3) O3-PbTiO3 single crystals under combined thermal and mechanical loading. Acta Mater..

[15] Luo J., Taylor S., Hackenberger W., Zhang S. Large field property assessment of Mn: PIN-PMN-PT crystals for high power transducers. Proceedings of the 2013 Joint IEEE International Symposium on Applications of Ferroelectric and Workshop on Piezoresponse Force Microscopy (ISAF/PFM).

[16] Luo J., Hackenberger W., Zhang S., Shrout T.R. The progress update of relaxor piezoelectric single crystals. Proceedings of the 2009 IEEE International Ultrasonics Symposium.

[17] Huo X., Zhang S., Liu G., Zhang R., Luo J., Sahul R., Cao W., Shrout T.R. (2013). Complete set of elastic, dielectric, and piezoelectric constants of [011] C poled rhombohedral Pb (In0.5Nb0.5) O3-Pb (Mg1/3Nb2/3) O3-PbTiO3: Mn single crystals. J. Appl. Phys..

[18] Gallagher J.A., Tian J., Lynch C.S. (2014). Composition dependence of field induced phase transformations in [0 1 1] C PIN–PMN–PT relaxor ferroelectric single crystals with d322 piezoelectric mode. Acta Mater..

[19] Sherlock N., Garten L., Zhang S., Shrout T.R., Meyer R.J. (2012). Nonlinear dielectric response in piezoelectric materials for underwater transducers. J. Appl. Phys..

[20] Snook K.A., Rehrig P.W., Hackenberger W.S., Jiang X., Meyer Jr R.J., Markley D. Advanced piezoelectric single crystal based transducers for naval sonar applications. Proceedings of the Smart Structures and Materials 2005: Active Materials: Behavior and Mechanics.

[21] Rehrig P., Snook K., Hackenberger W.S., Meyer Jr R.J., Markley D. 5D-5 tailored single crystal orientations for improved tonpilz transducer performance. Proceedings of the 2006 IEEE Ultrasonics Symposium.

[22] Zhang K., Chen Y., Peng K. The study of 32-mode single crystal longitudinal transducer. Proceedings of the 2016 IEEE/OES China Ocean Acoustics (COA).

[23] Jones D.F., Lewis D., Reithmeier C., Brownell G. Barrel-stave flextensional transducers for sonar applications. Proceedings of the International Design Engineering Technical Conferences and Computers and Information in Engineering Conference.

[24] Teng D., Liu X., Gao F. (2021). Effect of concave stave on class I barrel-stave flextensional transducer. Micromachines.

[25] Shao T., Du H., Ma H., Qu S., Wang J., Wang J., Wei X., Xu Z. (2017). Potassium–sodium niobate based lead-free ceramics: Novel electrical energy storage materials. J. Mater. Chem. A.

[26] Jung H.-B., Kim J.-W., Lim J.-H., Kwon D.-K., Choi D.H., Jeong D.-Y. (2020). Energy storage properties of blended polymer films with normal ferroelectric P (VDF-HFP) and relaxor ferroelectric P (VDF-TrFE-CFE). Electron. Mater. Lett..

[27] Perumal R.N., Athikesavan V. (2020). Structural and electrical properties of lanthanide-doped Bi0. 5 (Na0. 80K0. 20) 0.5 TiO3–SrZrO3 piezoelectric ceramics for energy-storage applications. J. Mater. Sci. Mater. Electron..

[28] Priya S., Viehland D., Carazo A.V., Ryu J., Uchino K. (2001). High-power resonant measurements of piezoelectric materials: Importance of elastic nonlinearities. J. Appl. Phys..

[29] Uchino K., Zheng J., Chen Y., Du X., Ryu J., Gao Y., Ural S., Priya S., Hirose S. (2006). Loss mechanisms and high power piezoelectrics. J. Mater. Sci..

[30] Westerhold S. (2022). Total Harmonic Distortion (THD) Analysis Utilizing the FFT Capabilities of Modern Digital Storage Oscilloscopes. https://zenodo.org/records/6969825.

[31] Neto L.B., Coelho P.H.G., de Mello J.C.C.S., Angulo-Meza L., Gomes E.G. Towards an intelligent system for monitoring electrical energy quality-Foundations and Motivations. Proceedings of the International Conference on Enterprise Information Systems.

[32] Je Y., Sim M.-S., Cho Y.-H., Lee W.-O., Lee S.-G., Lee J.-M., Seo H.-S. (2021). Characterization of [011] Poled Pb(In1/2Nb1/2)O3-Pb(Mg1/3Nb2/3)O3-PbTiO3 Single Crystals by Resonance Method. J. Korean Inst. Electr. Electron. Mater. Eng..

[33] Kwon B., Pyo S., Je Y., Seo H.-S., Lee S., Roh Y. (2024). Precise evaluation of the material constants of [011]-poled mangan-doped 0.32 Pb (In1/2Nb1/2) O3-0.39 Pb (Mg1/3Nb2/3) O3-0.29 PbTiO3 single crystals. AIP Adv..

[34] Zhang X., Lin X., Guo R., Yu F., Yang C., Huang S. (2023). Influence and mechanism of radial prestress on the electro-acoustic performance of spherical transducers. Sens. Actuators A Phys..

[35] Covaci C., Gontean A. (2020). Piezoelectric energy harvesting solutions: A review. Sensors.

